# Feeding and Oviposition Behaviour of *Trioza erytreae* (Hemiptera: Triozidae) on Different Citrus Rootstock Material Available in Europe

**DOI:** 10.3390/insects12070623

**Published:** 2021-07-08

**Authors:** Estrella Hernández-Suárez, Laura Suárez-Méndez, Moneyba Parrilla, Juan M. Arjona-López, Aurea Hervalejo, Francisco J. Arenas-Arenas

**Affiliations:** 1 Departamento de Protección Vegetal, Instituto Canario de Investigaciones Agrarias (ICIA), Ctra. El Boquerón s/n, 38270 La Laguna, Spain; ehernand@icia.es (E.H.-S.); lsuarez@icia.es (L.S.-M.); mparrilla@icia.es (M.P.); 2 Department of Agri-Food Engineering and Technology, Andalusian Institute of Agricultural and Fisheries Research and Training (IFAPA), “Las Torres” Center, Ctra. Sevilla-Cazalla de la Sierra km. 12.2, 41200 Alcalá del Río, Spain; aurea.hervalejo@juntadeandalucia.es (A.H.); fjose.arenas@juntadeandalucia.es (F.J.A.-A.)

**Keywords:** African citrus psyllid, citrus rootstock, host choice behavior, Huanglongbing, survival, sustainable pest management

## Abstract

**Simple Summary:**

The Mediterranean Basin is the second largest citrus-producing region in the world. Huanglongbing (HLB) is the most important and devastating citrus disease worldwide, the causal agents of which are three bacteria species that belong to the genus *Candidatus* Liberibacter. The main transmission of these three bacteria is by two psyllid vectors, *Diaphorina citri* and *Trioza erytreae*. *Trioza erytreae* is a specific pest of plants from the *Rutaceae* family, which includes cultivated citrus. This insect has recently been detected in Mediterranean Basin countries, such as Spain and Portugal, but none of the causal agents of HLB have been described as infecting citrus plants in this region. The potential risk of HLB emergence has increased concern in the Spanish and Portuguese citrus industries, which require novel methods for controlling/eradicating populations of *T. erytreae* that are environmentally sound under new pesticide restrictions from European Union authorities. Hence, psyllid rootstock feeding preferences could play an important role in pest management. Thus, different citrus rootstocks have been tested for feeding, oviposition, and survival of *T. erytreae*. This study reports that the most commonly used citrus rootstocks (Carrizo citrange and *Citrus macrophylla*) are the favorite choices for the development of *T. erytreae*, and by contrast *Poncirus trifoliata* is the least suitable for this insect vector.

**Abstract:**

*Trioza erytreae* (Del Guercio, 1918) (Hemiptera: Triozidae) is a vector of *Candidatus* Liberibacter spp., the causal agent of Huanglongbing disease (HLB). This study evaluates the preference of *T. erytreae* in different citrus seedlings. Thus, six different non-grafted citrus rootstocks were used for these experiments: (a) Carrizo citrange; (b) *Citrus macrophylla*; (c) ‘Cleopatra’ mandarin; (d) Forner-Alcaide No. 5; (e) Forner-Alcaide No. 517, and (f) *Poncirus trifoliata* (‘Flying Dragon’). The behaviour and survival of this psyllid was evaluated through the feeding preference of *T. erytreae* adults for different rootstocks (in a choice trial under greenhouse conditions) and oviposition and survival of *T. erytreae* adults on the different citrus material (in a no-choice trial under laboratory conditions). *Trioza erytreae* showed a clear preference for hosting and feeding on *C. macrophylla,* and Carrizo citrange was the most suitable rootstock for insect reproduction and survival followed by *C. macrophylla*. Conversely, *Poncirus trifoliata* was the least attractive rootstock to *T. erytreae* adults in the greenhouse trial and led to significantly lower *T. erytreae* survival. Our results suggest that conventional citrus rootstocks, such as Carrizo citrange and *C. macrophylla,* could increase *T. erytreae* populations.

## 1. Introduction

The Mediterranean Basin, with production exceeding 26 million tons, is the second largest citrus-producing region worldwide after China [1]. The African citrus psyllid, *Trioza erytreae* (Del Guercio, 1918) (Hemiptera: Triozidae), is one of the insect vectors of Huanglongbing (HLB), or citrus greening disease [2]. Currently, HLB is described as one of the most devastating citrus diseases worldwide [3,4]. HLB is associated with three non-culturable and Gram-negative bacterial species, *Candidatus* Liberibacter asiaticus (Jagoueix, Bové and Garnier) (CLas), Ca. Liberibacter americanus (Teixeira, Saillard, Eveillard, Danet, da Costa, Ayres and Bové) (CLam), and Ca. Liberibacter africanus (Jagoueix, Bové and Garnier) (CLaf) [5,6,7,8,9]. Specifically, *T. erytreae* has been reported to be capable of transmitting CLaf and CLas under natural conditions [5,10].

CLaf is heat sensitive and adapts to temperatures below ~30 °C [11]. CLas is reported as the most destructive and heat-tolerant strain, and can also be transmitted by *Diaphorina citri* (Kuwayama) (Hemiptera: Liviidae) [12]. CLam was first described in the state of Sao Paulo (Brazil) in 2004 and in the state of Texas (USA) in 2013 [13,14].

The joint natural distribution of *T. erytreae* and CLaf is mainly in Africa, where occurrences have been reported in several countries (Cameroon, Ethiopia, Somalia, Kenya, Tanzania, Malawi, Rwanda, Zimbabwe, South Africa, and Swaziland; the islands of Comoros, Saint Helena, Mauritius, Reunion, and Madagascar) and the Arabian Peninsula (Saudi Arabia and Yemen). Additionally, *T. erytreae* has also been reported in other African countries, such as Sudan, Eritrea, Uganda, Zambia, Angola, the Democratic Republic of the Congo, and in the islands of Sao Tome and Principe [15]. This insect vector was first detected in Europe in the archipelago of Madeira (Portugal) in 1994 [16] and in the Canary Islands (Spain) in 2002 [17]. In 2014, *T. erytreae* was first detected in mainland Europe in Galicia (Spain) and in Porto (Portugal) in 2015 [18], from where it spread throughout the western Atlantic coast, ranging from Cedeira in A Coruña (Spain) to Pontes/Setúbal in Portugal, which has raised alarm bells in the main citrus orchards in southern Spain and Portugal [19,20]. More recently, *T. erytreae* has been described in northern Spain (Asturias, Cantabria and Basque Country) [18]. To date, no species of Ca. Liberibacter spp. have been detected in Mediterranean Basin countries [21].

Although *T. erytreae* has been described as a weak flyer [22], it can be a highly invasive insect [23]. Under natural conditions, *T. erytreae* is relatively stationary and disperses only weakly; however, when conditions become adverse, it can show quite a different behaviour, with a great dispersal capacity, allowing individuals to locate new locations for egg laying [24]. The presence of *T. erytreae* can easily be detected in the field because their nymphs are found only on the underside of the leaves, where their activity typically leads to the formation of galls, each corresponding to a nymphal nest. Each nest consists of a globular distortion on the upper side of the leaf corresponding to a concave hollow on the lower side, which is inhabited by the nymphs until their development is complete. The adults then leave the nests, but the empty hollows remain clearly visible, allowing for an early diagnosis of the presence of *T. erytreae* [19,20,25].

In addition, *T. erytreae* is a specific pest of plants in the *Rutaceae* family, which includes both wild and cultivated citrus [15]. This insect can feed on at least 18 plant species, but egg laying and nymphal development is restricted to 15 and 13 species, respectively [26]. Common citrus *T. erytreae* host plants include *Citrus limon* (L.) Osbeck, *Citrus jambhiri* Lush., *Citrus medica* (L.), *Citrus aurantifolia* Christ., *Citrus sinensis* (L.), *Citrus reticulata* Blanco, *Citrus paradisi* Macfad., *Citrus maxima* (Burm.) Merr., and *Citrus unshiu* Swingle [26,27]. Adults and nymphs breed and feed exclusively on phloem sap in young leaves and shoots, causing damage, with typical symptoms including yellow and irregular shoots and open gall-like structures on citrus crop leaves [28,29,30].

Currently, Carrizo citrange (*Citrus sinensis* L. Osb. x *Poncirus trifoliata* L. Raf.) is the most widely used citrus rootstock in the Mediterranean Basin. While this rootstock is cold tolerant, it is highly sensitive to salinity [31,32,33]. Other conventional citrus rootstocks, such as ‘Cleopatra’ mandarin (*Citrus reshi* Hort. ex Tan.) and *Citrus macrophylla* Wester, exhibit other abiotic and biotic limitations, such as root asphyxia and susceptibility to nematodes (*Tylenchulus semipenetrans* Hodges).

Thus, different breeding programmes have resulted in many interesting citrus rootstocks with a substandard or semi-dwarfing character. Such is the case of Forner-Alcaide No. 5 (‘Cleopatra’ mandarin x *Poncirus trifoliata* (L.) Raf.) and Forner-Alcaide No. 517 (‘King’ mandarin x *Poncirus trifoliata* (L.) Raf.). Both rootstocks are resistant to the citrus tristeza virus (CTV), tolerant to citrus nematode and oomycetes, such as *Phytophthora* spp. [34,35,36], and further, as opposed to Carrizo citrange, they display a high salinity tolerance and a better response to active limestone. HLB tolerance has been identified in certain citrus species and citrus-related genera, including trifoliate orange, *Poncirus trifoliata* (L.) Raf. [37,38,39,40,41]. Studies have reported a relationship between the resistance or tolerance of plant material to disease and the psyllid colonisation ability. Thus, Ramadugu et al. [39] conducted a study assessing the long-term resistance to HLB by citrus relatives, which attributed the tolerance observed in different accessions to a number of factors, including the psyllid colonisation ability. Prior research has described that resistance to pest attacks is induced by different citrus rootstocks [42,43]. Thus, the rootstock can impart physicochemical changes to the commercial cultivar against insect pests [44]. In this sense, other surveys have studied the host preference of the other psyllid vector (*D. citri*) in different rootstocks [45,46,47]. The most recent study, which researched the preference of *D. citri* on three commonly cultivated rootstocks, found the rootstock Carrizo citrange is highly vulnerable to this insect vector [47]. However, to our knowledge no prior studies have carried out the rootstock suitability of *T. erytreae*.

Even in a scenario in which HLB has not yet arrived in the Iberian Peninsula, choosing a suitable rootstock may be a helpful and sustainable method to prevent and manage HLB and *T. erytreae* populations [48]. Reducing the shoot flushing period has been reported as a strategy for creating unfavourable conditions for *T. erytreae*, as this insect hosts young shoots [49]. Thus, factors impacting the flushing cycle of trees, such as rootstocks [50], may significantly affect the development of psyllid populations in field conditions and have repercussions on insect control strategies. As such, this sustainable practice would help decrease pest incidence in an integrated management programme, reducing the use of phytosanitary products due to environmental concerns and pesticide restrictions in Europe [51].

The main aim of this study was to assess the colonisation behaviour of *T. erytreae* in different non-grafted citrus rootstocks available in the Spanish citrus industry, by characterising *T. erytreae* survival, development, oviposition and preference for different plant materials under both laboratory and greenhouse conditions.

## 2. Materials and Methods

To assess the suitability of plant material for *T. erytreae*, the behaviour and survival of this psyllid was evaluated, and two types of experiments were carried out to test the following: (i) the feeding preference of *T. erytreae* adults for different rootstocks (in a choice trial under greenhouse conditions), and (ii) the oviposition and survival of *T. erytreae* adults reared on different citrus material (in a no-choice trial under laboratory conditions).

### 2.1. Plant Material and Experimental Design

Six different non-grafted citrus rootstocks were used for our experiments: (a) Carrizo citrange; (b) *C. macrophylla*; (c) ‘Cleopatra’ mandarin; (d) Forner-Alcaide No. 5; (e) Forner-Alcaide No. 517, and (f) *P. trifoliata* (‘Flying Dragon’). Plant material used was supplied by the Andalusian Institute of Agricultural and Fisheries Research and Training (IFAPA) from a nursery in Seville, Spain.

All plant material was grown individually in plastic seedling pots (20 × 30 cm) containing a mixture of dung, compost and vermiculite in equal parts as the substrate. Plants were watered twice a week and fertilised with an N-P-K solution once a week. Plants were kept in a greenhouse until they reached a height of approximately 1 m. Two-year-old pesticide-free plants were used in the experiments. Around one month before the experiments began, all the plants from these rootstocks had all their growing tips cut off to stimulate sprout meristem shoots, which is required for *T. erytreae* feeding, oviposition and survival.

### 2.2. Source of Insects

*Trioza erytreae* individuals were originally collected from a commercial sweet orange orchard located in the municipality of Tegueste (Tenerife, Canary Islands). This insect stock colony was maintained on two-year-old pesticide-free citrus plants: lemon (cv. Eureka grafted on *C. macrophylla*) and sweet orange (cv. Lane Late IVIA 188 grafted on Carrizo citrange). Plants were grown in pots (Ø = 20 cm) under controlled conditions (20 ± 5 °C, RH > 70%, 16:8 h (L:D) photoperiod), and they were regularly pruned to stimulate the emergence of new shoots. Adults from this colony were tested by the diagnostic standard PM 7/121 (1) of the European and Mediterranean Plant Protection Organization [52], firstly with the screening test of Bertolini et al. [53] and secondly, for those samples that were positive in the first step, using the species-specific primers and Taq-Man probe for Claf, Clas and Clam described by Li et al. [54].

The *T. erytreae* rearing system consisted of one screen cage for oviposition and another for nymphal development. Ten plants were placed in the egg-laying cage (2.90 m × 2.70 m × 4.50 m). One hundred insects were used for oviposition at a sex ratio of 1:1. Two days later, adults were removed with a handheld aspirator and the plants were transferred into the nymphal development cage (140 × 100 × 100 cm).

### 2.3. Feeding Preference of T. erytreae Adults for Different Rootstocks

This study was carried out over two consecutive years (2018 and 2019) in a 256 m^2^ polycarbonate greenhouse belonging to the Canarian Institute for Agricultural Research (ICIA) located at the Isamar experimental station in the Valle Guerra area, in the municipality of La Laguna (Tenerife, Spain). Three antithrips mesh walk-in cages of 2.90 × 2.70 × 4.50 m were raised within the greenhouse. Twelve plants were arranged regularly in each cage in a randomised design, with two plants of each citrus rootstock per cage.

The trial started when all the plants had at least two shoots, each 4 to 5 cm in length. Prior to the experiment, a detailed inspection of the plant material was performed to rule out any previous contamination by *T. erytreae* that could interfere with experimental results. One hundred *T. erytreae* adults were released in each cage in 2018 and 200 adults were released in each cage in 2019; the same experimental conditions and plants were applied in both years. For these plants a long period time was allowed between trials and the plants were pruned twice before the 2019 trial. Evaluations were then carried out, which involved counting the number of adults per plant at 3, 7, 14, and 21 days after the release of the insects. Climatic conditions were recorded throughout the test period using an Omega-92 data logger (Omega Engineering Inc., Norwalk, CT, USA). The recorded climate data consisted of average daily temperature and average daily relative humidity. During the 2018 trial period, the average, maximum and minimum temperatures recorded were 24.7, 41.6 and 16.1 °C, respectively, and the average, maximum and minimum relative humidities recorded were 67.6, 99.2 and 25.4%. During the 2019 trial period, the average, maximum and minimum temperatures recorded were 24.3, 35.7 and 17.9 °C, respectively, and the average, maximum and minimum relative humidities recorded were 67.7, 88.8 and 39.0%.

### 2.4. Oviposition and Survival of T. erytreae

The *T. erytreae* non-choice oviposition and survival trials were carried out in 2019 in a walk-in insect rearing chamber under controlled conditions, with a temperature of 20 ± 5 °C, a relative humidity of 70 ± 10%, and a photoperiod of 16:8 h (L:D). To assess oviposition, two mature adult *T. erytreae* couples were released per shoot (confined within a muslin bag). The experimental design was fully randomised, with sixteen repetitions (each consisting of two mature adult couples) per treatment (rootstock), considering the shoot as the experimental unit and selecting four shoots per plant in a total of four plants. To ensure consistency in the suitability of shoots among the rootstocks, young shoots of similar lengths (approximately 4 to 5 cm) were chosen. After 72 h, the number of eggs per shoot was counted using a stereoscopic microscope, the the length of the shoots was measured, psyllid adults were sexed, and the repetitions in which any of the gravid females had died were discarded.

To assess survival, a group of ten *T. erytreae* adults (5 ♂ and 5 ♀) less than 72 h old (collected from the insect colony) were confined to a whole plant in mesh sleeve cages within the rearing chamber. The experimental design was fully randomised, in which each plant was the experimental unit, with four plant replicates per treatment (rootstock), and five insects couples were inoculated in each confined plant. Survival was evaluated at 1, 3, 5, 7, 10, 17, 24, and 31 days after confinement. The percentage of *T. erytreae* adult survival was calculated using the following equation: *T. erytreae* adult survival = (T_n_/T_0_) × 100; where: T_n_ = number of *T. erytreae* adults per assessment period, rootstock and corresponding confined plant; T_0_ = number of *T. erytreae* adults at the beginning of the experiment (period 0) per rootstock and confined plant.

### 2.5. Data Analyses

All raw values obtained in this study from each experiment performed were analyzed by one-way ANOVA and the LSD-Fisher test (*p* < 0.05) [55] with the free software R version 4.0.2 [56] through the “agricolae” package [57].

## 3. Results

### 3.1. Feeding Preference of T. erytreae Adults for the Different Rootstocks

**Figure 1:**
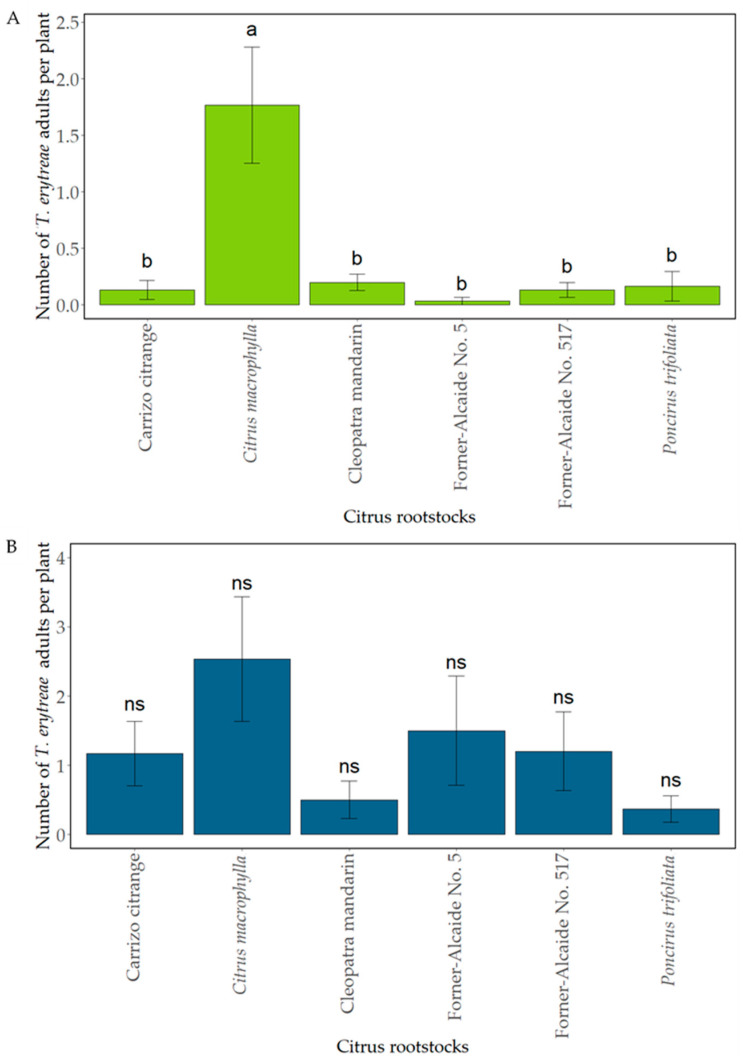
Mean of *T. erytreae* adults per plant ± standard error (SE) on the different non-grafted citrus rootstocks tested during the 21-day free-choice feeding experiment, performed under greenhouse conditions, in each year tested. (**A**) 2018. (**B**) 2019. Values in columns with different letters are significantly different among the rootstocks assayed per year by LSD-Fisher test (*p* < 0.05). *Poncirus trifoliata*: *Poncirus trifoliata* (‘Flying Dragon’); ns: no significant differences.

In the 2018 greenhouse trial, significantly different responses were obtained among the rootstocks assayed (F_5,30_ = 9.03; *p* < 0.001). The results showed that the highest number of adults per plant was reached on *C. macrophylla*, with significantly higher numbers of psyllids than on the other rootstocks, which did not differ significantly amongst themselves, with average means ranging from 0.2 to 0.03 adults per plant for ‘Cleopatra’ mandarin and Forner-Alcaide No. 5, respectively (Figure 1A).

As to the 2019 experiment, no significant differences were obtained among the rootstocks assayed (F_5,30_ = 1.77; *p* = 0.15). However, *C. macrophylla* again showed the highest number of adults, followed by Forner-Alcaide No. 5. The lowest rate was achieved in *P. trifoliata* (Figure 1B).

### 3.2. Oviposition and Survival of T. erytreae on the Different Rootstocks

**Figure 2:**
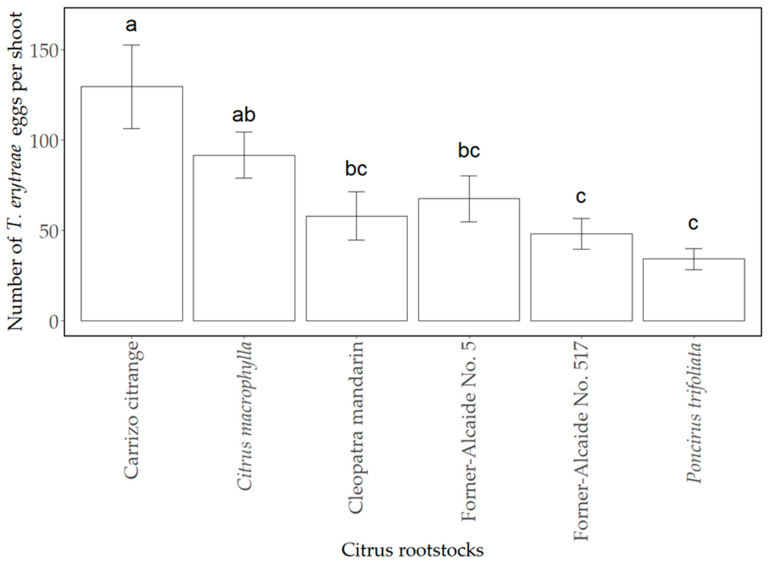
Mean of *T. erytreae* eggs per shoot ± standard error (SE) in the different non-grafted rootstocks tested after the 72-h no-choice laboratory trial. Values in columns with different letters indicate statistical differences among the rootstocks by LSD-Fisher test (*p* < 0.05). *Poncirus trifoliata*: *Poncirus trifoliata* (‘Flying Dragon’).

Overall, *T. erytreae* oviposition per shoot was significantly different among the rootstocks assayed (F_5,92_ = 6.32; *p* < 0.001). The highest value of oviposited eggs per shoot was reached in Carrizo citrange, followed by *C. macrophylla*, although the difference was not significant when compared with the highest value. On the contrary, the lowest significant oviposition rate was achieved on *P. trifoliata*, which was followed by Forner-Alcaide No. 517, ‘Cleopatra’ mandarin, and Forner-Alcaide No. 5 (in increasing order), without statistical differences compared with the response of *P. trifoliata* (Figure 2).

*Trioza erytreae* adult survival percentages differed among the rootstocks assayed according to the assessment period. The survival response for the rootstocks assayed showed similar results, without significant differences among the assessment periods of the first assessment day (Day 1; F_5,18_ = 2.32; *p* = 0.086), the tenth assessment day (Day 10; F_5,18_ = 1.98; *p* = 0.13), and the seventeenth assessment day (Day 17; F_5,18_ = 2.33; *p* = 0.08). In the latter two (Day 10 and Day 17), Carrizo citrange reported the highest survival values, while Forner-Alcaide No. 5 displayed the lowest values. On the other hand, on the first assessment day (Day 1), *C. macrophylla* and Forner-Alcaide No. 517 showed the highest and lowest survival rates, respectively. Similarly, during the third assessment day, *C. macrophylla* and Forner-Alcaide No. 517 showed the highest and the lowest survival rate, respectively, with significant differences (F_5,18_ = 4.17; *p* = 0.01); whereas the survival response for Forner-Alcaide No. 5 and *P. trifoliata* did not show statistical differences when compared with Forner-Alcaide No. 517. On Day 7, the highest and the lowest *T. erytreae* adult survival rates were recorded by ‘Cleopatra’ mandarin and Forner-Alcaide No. 5, respectively, with significant differences (F_5,18_ = 3.28; *p* = 0.028). Forner-Alcaide No. 517 and *P. trifoliata* displayed a survival response similar to that of Forner-Alcaide No. 5, without statistical differences. Finally, Carrizo citrange reported the highest significant adult survival rate in the two last assessment days (Day 24; F_5,18_ = 3.06; *p* = 0.036, and Day 31; F_5,18_ = 3.03; *p* = 0.037), on which *C. macrophylla* and *P. trifoliata* showed the lowest rates on the twenty-fourth assessment day (Table 1).

## 4. Discussion

Studies have reported that *T. erytreae* can reproduce and survive, colonising a variety of citrus [58,59], which has also been described for another HLB vector, *D. citri* [60,61]. However, *T. erytreae* reproduces exclusively in young shoots, which must not reach the state of maturity before nymphal development is completed, as this leads to nymph death [29,49]. Therefore, the rate at which shoots sprout and their structural characteristics are essential to the development of *T. erytreae* populations [48], which suggests that factors influencing this rate will affect the development of pest populations.

Tamesse and Messi [62] compared around 30 citrus cultivars in terms of their vulnerability under field conditions in Cameroon, describing preference for certain cultivars over others and recording *T. erytreae* feeding, development and egg laying. Similarly, we have studied the exposure of non-grafted rootstocks to *T. erytreae*, recording different parameters, such as feeding preference, oviposition, and survival. Non-grafted *C. macrophylla* rootstock appears to be more vulnerable to *T. erytreae* adult feeding behaviour than the remaining tested rootstocks. On the contrary, Forner-Alcaide No. 5 and *P. trifoliata* were the least preferred for *T. erytreae* feeding. Thus, Forner-Alcaide No. 5 is an interesting rootstock due to its suitable agronomic and quality characteristics [36,63] induced in the grafted cultivar. Moran [29] studied the antenna structure of this psyllid, demonstrating the role of antennal chemoreceptors in the detection of secondary plant compounds and indicating that this could play a role in the choice of host plant.

However, the oviposition laboratory trial provided evidence that among the rootstocks tested, Carrizo citrange is the most suitable rootstock for *T. erytreae* oviposition. A recent study by Urbaneja-Bernat et al. [47] indicated that the Carrizo citrange rootstock is also highly vulnerable to *D. citri.* In their greenhouse experiments, this commonly used citrus rootstock proved to be the most suitable for the development and reproduction of *D. citri* among the three rootstocks tested, showing higher oviposition and egg hatching rates. For this reason, it is necessary to secure alternative rootstocks for famers to increase plant material diversity, including options to replace rootstocks, such as Carrizo citrange, when appropriate.

According to our results, *T. erytreae* reported the lowest host preference on *P. trifoliata* throughout the trials. In a free-choice situation this rootstock showed low colonisation by *T. erytreae*, with a lower number of adult visits per plant. On the other hand, in a non-choice assay, it was the least suitable for oviposition and recorded a low *T. erytreae* survival rate. In this sense, *P. trifoliata* could be an interesting alternative to Carrizo citrange. *Poncirus trifoliata* has been used as a parent in different citrus rootstock breeding programmes because it induces sound productivity and fruit quality. Furthermore, it shows resistance to citrus nematodes and root asphyxia. More recently, this citrus rootstock has sparked great interest, since *P. trifoliata* and trifoliate citrus rootstock hybrids have been reported as being tolerant to HLB [37,38,64]. Other citrus rootstocks, such as Forner-Alcaide No. 5, Forner-Alcaide No. 517 and ‘Cleopatra’ mandarin are interesting alternatives to Carrizo citrange, with less oviposition and a lower *T. erytreae* survival rate than Carrizo citrange. In addition, it is less vulnerable to the *T. erytreae* adult feeding behaviour than *C. macrophylla* in areas with salinity and/or active limestone conditions [34,35,65].

Nava et al. [66] mentioned that *D. citri* development and survival is lower in the ‘Sunki’ tangerine (*Citrus sunki* (Hayata) hort ex. Tanaka). Additionally, Tsagkarakis and Rogers [45] reported that these parameters also decrease for ‘Cleopatra’ mandarin, while Westbrook et al. [67] also showed that *D. citri* avoids colonising *P. trifoliata*. Our results for *T. erytreae* on non-grafted ‘Cleopatra’ mandarin and *P. trifoliata* rootstocks are consistent to those previously reported for *D. citri*. On these two rootstocks, together with the Forner-Alcaide No. 517 and Forner-Alcaide No. 5 rootstocks, *T. erytreae* showed lower survival and oviposition when compared to the Carrizo citrange rootstock. Therefore, these citrus rootstocks which displayed the least *T. erytreae* host preference should be taken into consideration for future citrus breeding programmes and nursery plant management. This will help to reduce *T. erytreae* populations and the risk of HLB emergence in disease-free areas.

## Figures and Tables

**Table 1:** Mean of *T. erytreae* adults survival (%) ± standard error (SE) in the six different rootstocks assayed for the different assessment periods.

Citrus Rootstock	*Trioza erytreae* Adults Survival (%)							
	Day 0	Day 1	Day 3	Day 7	Day 10	Day 17	Day 24	Day 31
Carrizo	100.00 ± 0.00 ns	91.25 ± 5.91 ns	83.13 ± 8.00 abc	80.63 ± 8.68 ab	65.63 ± 4.83 ns	60.00 ± 7.07 ns	31.25 ± 8.75 a	17.50 ± 8.54 a
*C. macrophylla*	100.00 ± 0.00 ns	96.88 ± 3.13 ns	93.75 ± 3.61 a	78.13 ± 3.13 ab	62.50 ± 8.84 ns	50.00 ± 8.84 ns	0.00 ± 0.00 b	0.00 ± 0.00 b
Cleopatra	100.00 ± 0.00 ns	92.50 ± 4.79 ns	89.38 ± 4.13 ab	86.88 ± 4.72 a	63.13 ± 4.72 ns	38.13 ± 13.36 ns	12.50 ± 9.46 ab	0.00 ± 0.00 b
FA5	100.00 ± 0.00 ns	92.50 ± 4.79 ns	75.00 ± 6.45 bcd	55.00 ± 9.57 c	37.50 ± 7.50 ns	20.00 ± 5.77 ns	5.00 ± 5.00 b	5.00 ± 5.00 b
FA517	100.00 ± 0.00 ns	78.13 ± 5.98 ns	65.63 ± 5.98 d	62.50 ± 8.84 bc	59.38 ± 7.86 ns	37.50 ± 8.84 ns	15.63 ± 9.38 ab	0.00 ± 0.00 b
*P. trifoliata*	100.00 ± 0.00 ns	81.25 ± 3.61 ns	68.75 ± 3.61 cd	59.38 ± 5.98 bc	53.13 ± 9.38 ns	37.50 ± 7.22 ns	0.00 ± 0.00 b	0.00 ± 0.00 b

Values with different letters indicate statistical differences among the rootstocks per assessment period by LSD-Fisher test (*p* < 0.05). ns: no significant differences. Day: Assessment day; Carrizo: Carrizo citrange; *C. macrophylla*: *Citrus macrophylla*; Cleopatra: ‘Cleopatra’ mandarin; FA5: Forner-Alcaide no. 5; FA517: Forner-Alcaide no. 517; *P. trifoliata*: *Poncirus trifoliata* (‘Flying Dragon’).

## Data Availability

Not applicable.
